# High shear *in situ* exfoliation of 2D gallium oxide sheets from centrifugally derived thin films of liquid gallium†

**DOI:** 10.1039/d1na00598g

**Published:** 2021-09-01

**Authors:** Kasturi Vimalanathan, Timotheos Palmer, Zoe Gardner, Irene Ling, Soraya Rahpeima, Sait Elmas, Jason R. Gascooke, Christopher T. Gibson, Qiang Sun, Jin Zou, Mats R. Andersson, Nadim Darwish, Colin L. Raston

**Affiliations:** Flinders Institute for Nanoscale Science and Technology, College of Science and Engineering, Flinders University Adelaide SA 5001 Australia kasturi.vimalanathan@flinders.edu.au colin.raston@flinders.edu.au; School of Science, Monash University Malaysia Jalan Lagoon Selatan, Bandar Sunway 47500 Selangor Malaysia; School of Molecular and Life Sciences, Curtin Institute for Functional Molecule and Interfaces, Curtin University Bentley Western Australia 6102 Australia; Flinders Microscopy and Microanalysis, College of Science and Engineering, Flinders University Bedford Park SA 5042 Australia; Centre for Microscopy and Microanalysis, The University of Queensland Brisbane QLD 4072 Australia; Materials Engineering, The University of Queensland Brisbane QLD 4072 Australia

## Abstract

A diversity of two-dimensional nanomaterials has recently emerged with recent attention turning to the post-transition metal elements, in particular material derived from liquid metals and eutectic melts below 330 °C where processing is more flexible and in the temperature regime suitable for industry. This has been explored for liquid gallium using an angled vortex fluidic device (VFD) to fabricate ultrathin gallium oxide (Ga_2_O_3_) sheets under continuous flow conditions. We have established the nanosheets to form highly insulating material and have electrocatalytic activity for hydrogen evolution, with a Tafel slope of 39 mV dec^−1^ revealing promoting effects of the surface oxidation (passivation layer).

## Abstract 2

Ultrathin gallium oxide sheets formed under continuous flow from gallium metal are highly insulating with electrocatalytic activity for hydrogen evolution.

## Graphical abstract

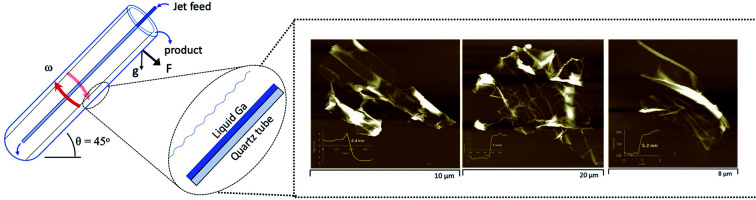

## Introduction

**Fig. 1:**
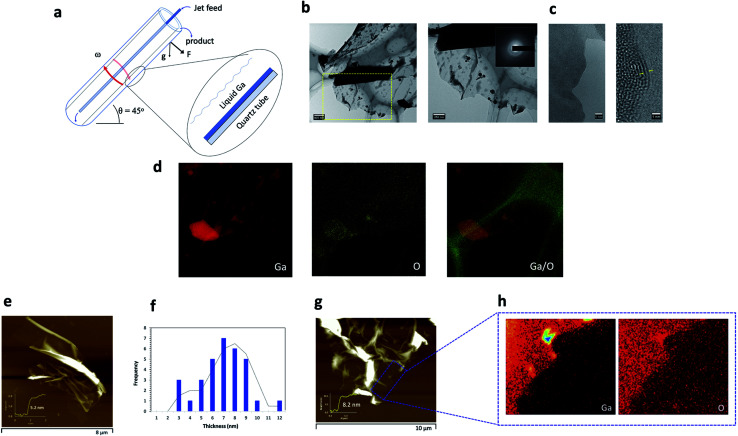
VFD mediated fabrication of ultrathin Ga_2_O_3_ sheets. (a) Schematic of the VFD illustrating its salient features. (b) Bright field TEM images of an ultrathin sheet. (c) HRTEM images illustrating the lattice spacing of the sheets of ∼0.25 nm. (d) EDS maps of the corresponding sheet in (b). (e) AFM height image of an ultrathin sheet. (f) An average thickness distribution plot based on AFM measurements and (g, h) EDX map of the ultrathin sheet confirming the presence of Ga and O on the surface of the area corresponding to the section in (g).

Over the past decade, 2D-nanomaterials have captured the attention of the scientific community, with their extraordinary properties and diversity in applications, seemingly having no boundaries.^1–3^ Nevertheless, there are shortcomings on gaining access to such material where scalability is addressed. This even applies to ubiquitous graphene, for the fabrication of high quality atomically thin sheets, in overcoming the strong van der Waals forces between the layers, and the limited dispersion prowess of the material in organic solvents. While graphene itself is a zero-band gap semiconductor and exhibits quasi metallic features, it exhibits limitations in applications primarily for use in devices and electronics.^4,5^ Having access to a library of 2D-nanomaterials with different properties offers scope for being able to tune the properties of the materials, and this has emerged as a driver for developing 2D-nanomaterials beyond graphene. A number of 2D materials that have recently emerged include transition metal dichalcogenides (TMDs), layered metal oxides, 2D inorganic compounds, hexagonal boron nitride (h-BN), layered double hydroxides (LDHs), black phosphorus (BP) and other elemental main group metals and metalloids.^5–15^ 2D metal oxides derived from liquid metals and/or eutectic melts of the metals, in particular, are envisaged to break new barriers, offering unique properties arising from their high specific surface area and atomic structure which directly influences electronic, optical and magnetic behavior, for potential translation into different applications.^16–26^ In addition, it is a significant advantage that any development as such needs to incorporate the principles of green chemistry to minimise the likelihood of any downstream issues, including adverse effects on the natural environment, with the process being readily scalable. We have recently explored the potential of mechanical energy induced within thin films of liquid in the variable angle vortex fluidic device (VFD), Fig. 1a, for the synthesis and manipulation of nanomaterials, with the effectiveness of this type of processing in exfoliation of 2D materials, including graphene, h-BN and black phosphorus.^27–29^ This approach has been successful for generating such material high in green chemistry credentials, without the need for surfactants and harsh chemicals, thereby minimising the waste stream, with the processing simple and low costing, using low molecular weight solvents and under continuous flow conditions. The angled VFD with an optimal tilt angle of 45° (maximum cross vector between centrifugal force and gravity) spun at high rotational speeds has remarkably diverse range of applications beyond the exfoliation of 2D materials, including scrolling of graphene directly from graphite flakes, laser assisted controlled slicing of carbon nanotubes, controlling chemical reactivity and selectivity, biocatalysis and probing the structure of self-organised systems,^30–36^ also incorporating green chemistry principles within the processing.

Herein, we report a simple biphasic method involving liquid gallium and an organic solvent for the *in situ* generation and exfoliation of Ga_2_O_3_ sheets, yielding extremely thin sheets with lateral dimensions that are of the lateral dimensions of the bulk gallium layer centrifugally thinned. Gallium metal is a liquid at room temperature (mp 29.7 °C) having negligible vapour pressure, with the solid state exhibiting both covalent and metallic bonding, and excellent electrical and thermal conductivity.^37^ Also important is its low toxicity making the melt an attractive alternative to toxic mercury and making it useful for a number of applications.^38–42^ Similar to other liquid metals and alloys, gallium features a self-limiting thin oxide layer under ambient conditions, which partly contributes to the excellent properties the liquid metal has to offer at the metal air interface.^43^ 2D Ga_2_O_3_ sheets and nanoparticles have been previously reported using a variety of synthetic routes with the most common method involving high intensive cavitational energy, the squeeze-printing process whereby liquid gallium is forced between two substrates, and a nebulization technique with all resulting in surface oxidation, affording a nanometer thin layer of the oxide.^20,23,43–45^ We hypothesised that in the VFD, this layer has the potential to be exfoliated in contact with a dynamic thin film of solvent, thereby exposing fresh metal for rapid oxidation under high mass transfer of oxygen into the film of metal, as a continuous flow exfoliation process, one layer of the 2D material at a time. Exfoliation of 2D material in the VFD is under controlled mechanical energy input in time and place and we also hypothesized that this would lead to uniform sized sheets of any exfoliated material. While metal oxide layers can be transferred to other surfaces,^22,43^ strategies for direct transfer into a solvent for fabricating designer material are wanting. Also noteworthy is that the mechanical energy in a liquid over the liquid metal in the VFD can overcome the otherwise formation of a rigid mechanical shell (oxide layer) around the droplet of liquid metal, thereby attenuating its exfoliation.

The VFD was the thin film platform of choice given its utility in the top-down manipulation of a diverse range of nanomaterials, having exquisite control of morphology, size and dimensions of particles size to suit specific applications, for either exfoliation or exfoliation with scrolling of the 2D material as discussed above. The key difference now is that the processing is essentially a biphasic system with the source of the 2D material being surface oxidised liquid metal. Biphasic processing in the VFD thus far has been around immiscible solvents including water, for fabricating nanomaterials^46,47^ and the separation of proteins.^48^ To put it into perspective, the VFD features a borosilicate or quartz glass tube rotating at high speed (usually rotated between 2000 and 9000 rpm) inclined relative to the horizontal position, which is typically optimal for a specific process at 45°. Liquid is continuously delivered to the bottom of the glass tube, which then forms a dynamic thin film, Fig. 1a. The VFD encompasses a number of unique operating features that allows for efficient mass transfer of gases, intense micromixing of the reaction mediums and rapid dissipation of heat within the system which in combination is highly effective for the exfoliation of the Ga_2_O_3_ nanosheets.

## Results and discussion

The design of our processing method was established based on systematically exploring the process parameters of the VFD (rotational speed, *ω*, and inclination angle, *θ*), the choice of solvent, temperature of the system and residence time of liquid entering the tube and exiting at the top. Gallium solid was crushed in a mortar and pestle to finer powder particles prior to immersing in 1 mL of *n*-propanol (10 mg mL^−1^). *n*-Propanol was the choice of solvent after careful optimisation with other polar and non-polar solvents within the green chemistry scope, also including dimethylformamide (DMF), toluene, *i*-propanol, methanol, *o*-xylene and mesitylene. The mixture was then heated on a hot plate to *ca.* 30 °C for ∼60 seconds to achieve a molten liquid which was then subsequently transferred to a VFD quartz tube (20 mm OD, 17.5 mm ID, 18.5 cm long). The VFD process was optimised by systematically varying the rotational speed (4000 rpm to 9000 rpm) and the inclination angle (0° to 90°), with the optimised conditions 7000 rpm and *θ* = 45° respectively. The optimised speed is consistent with mixing times experiments undertaken to gain insight into the nature of the fluid dynamics within the thin film.^49^ We observed a minimum time taken for *n*-propanol to mix and form a uniform film at the 7000 rpm speed (Fig. S1†) which corresponds to the presence of beneficial Coriolis and Faraday wave effects in the thin film.^49^ We also carried out a control experiment using an *in situ* heating jacket, a ‘plug and play’ attachment on the VFD (Fig. S2†) and heated the system at 30 °C and 50 °C at the abovementioned optimised rotational speed and tilt angle. The heating jacket was used to avoid prior heating of gallium metal on a hotplate. However, no sheets were observed using this approach. To address scalability of the process, we translated these above conditions into a continuous flow processing platform, with the liquid gallium (200 mg) placed at the base of the quartz VFD tube with then injection of *n*-propanol at an optimised flow rate of 0.5 mL min^−1^. The gallium oxide sheets were produced in high yield (∼60%) following recycling through of the solvent, *n*-propanol. Here we established proof of concept of the potential for scaling up using parallel arrays of VFDs, coupled with recycling of the solvent and the gallium metal, or the use of the larger diameter, 50 mm VFD. Thus, heating the gallium metal to a molten liquid on a hot plate at 30 °C is important prior to processing in the VFD. The temperature of the hot plate was pivotal, with temperatures >30 °C showing no evidence of sheets being formed, and with temperatures below the melting point of gallium also ineffective. After 30 minutes of processing, the sheets were collected, centrifuged (*g* = 3.22) to remove any contamination and large particles/solids of unreacted gallium within the sample, and then dried for further characterization. Scanning electron microscopy (SEM) and atomic force microscopy (AFM) were initially used to establish the size, morphology and thickness of the sheets. Transmission electron microscopy (TEM), high resolution transmission electron microscopy (HRTEM), Raman spectroscopy, scanning auger, energy dispersive X-ray (EDX) mapping, X-ray photoelectron spectroscopy (XPS), and X-ray powder diffraction (XRD) analysis were then used to further understand the nature of the 2D-sheets.

Gaining insight into the mechanism of formation and surface properties of these ultrathin sheets, involved the use of AFM tapping mode and TEM and HRTEM on samples deposited directly on SiO_2_/Si wafers and on holey carbon grids respectively. The ultrathin sheets were observed to be thin with wrinkles on the surface. TEM/EDS (energy dispersive X-ray mapping) and SEM/EDX mapping were used to determine the elemental composition of the sheets, establishing the presence of Ga and O (Fig. 1h) whilst HRTEM of the sheets showed poor crystallinity with a *d*-spacing of 0.25 nm and amorphous characteristics (Fig. 1c).^50^ Electron diffraction of the ultrathin sheets (inset Fig. 1b) showed low intensity diffraction rings which is consistent with the HRTEM images, with the sheets having low crystallinity and partial amorphous characteristics. The oxidised gallium at the surface of the ultrathin sheets (Fig. 1) arises from processing under ambient conditions where there is high mass transfer of oxygen into the thin film of *n*-propanol in contact with the thin layer of gallium centrifugally pinned against the wall of the VFD tube. As previously noted, most liquid metals feature such a self-limiting oxide layer under ambient conditions. The high shear stress generated in the VFD at 7000 rpm and 45° tilt angle is effective in ‘peeling off’ individual layers of ultrathin Ga_2_O_3_ sheets in contact with liquid gallium through dispersion forces, with subsequent exposed gallium metal then forming another layer of oxidized metal, which is also exfoliated in the same way, and so on. The peeling of the sheets from the surface of the gallium is facilitated by upward flow in the spinning top high mass transport topological fluid flow μm in diameter or less.^46^ This flow was established in the VFD from moulding of bismuth metal (melting point 271.4 °C) which melts on the surface of the tube at room temperature by the mechanical energy induced by down flow of the spinning top flow.^46^

AFM height images established the average thickness of the exfoliated sheets at ∼5–6 nm (calculated from an average distribution of 50 sheets) (Fig. 1e and g). Given that a single oxide layer of Ga_2_O_3_ has a thickness ∼1 nm,^50^ the exfoliated Ga_2_O_3_ sheets generated in the VFD consist of approximately 5 to 6 layers of the oxide material. The rotational speed of 7000 rpm and 45° inclination angle was critical for high yield exfoliation, with all other combinations studied affording either no sheets or much lower yield, and thus the residence time and shear stress are critically important. The low crystallinity and partial amorphous characteristics, as observed from the HRTEM images, Fig. 1c, is related to the high rotational speed contributing towards the shorter residence time for the gallium to be within the reaction system and therefore limiting time for reorganisation of the crystal structure. This is consistent with literature whereby unalloyed gallium sheets tend to show amorphous characteristics specifically when fabricated over short time scales.^43^ Raman mapping of an ultrathin sheet, (Fig. S3†), was carried out for bulk gallium (black line), as well as for Ga_2_O_3_ sheets (blue line). The bulk gallium gave a characteristic band at approximately 300 cm^−1^ corresponding to the symmetrical stretching of pairs of gallium atoms.^51,52^ The Raman spectra of the generated ultrathin sheets in the present study have several weak and broad bands attributed to the bending and stretching of the Ga–O bond.^52^ In addition, there is a strong broad signal between 50 cm^−1^ and 400 cm^−1^ (Fig. S3†). The typical characteristic band around 300 cm^−1^ for gallium metal was not observed, possibly due to the low structural order of the Ga_2_O_3_ lattice.^52^ A slight background signal was also observed in the Raman spectra of Ga_2_O_3_ which could be due to luminescence effects.^52^

**Fig. 2:**
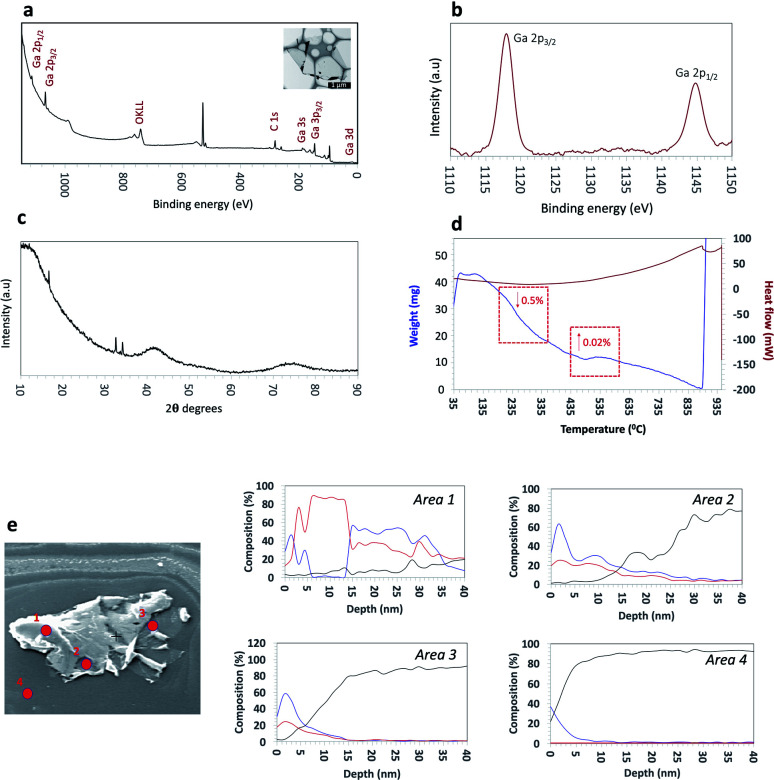
Surface characterization of the ultrathin Ga_2_O_3_ sheets. (a) XPS survey spectra of the Ga_2_O_3_ sheets. (b) High resolution XPS spectra showing two intense peaks at binding energies of 1145.03 eV and 1118.13 eV, which correspond to 2p_3/2_ and 2p_1/2_ states, respectively of the Ga_2_O_3_ sheets. (c) XRD pattern of the Ga_2_O_3_ sheets (*λ* = 1.79 Å). (d) Thermal analysis using TGA/DSC at 10 °C of temperature increase under N_2_ and (e) Auger depth profile analysis of an ultrathin Ga_2_O_3_ sheet (SEM image) and the corresponding depth profile graphs for 4 selected points (area 1–4) (figure legend: 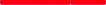 Ga;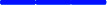 O; 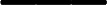 Si).

XPS measurements were carried out to further understand the surface composition and chemical states of the synthesized Ga_2_O_3_ sheets. The survey spectra showed the presence Ga, O and traces amount of carbon in the sample. The high-resolution spectra of the Ga 2p region afforded a doublet, Ga 2p_3/2_ and Ga 2p_1/2_ with the positions of the Ga_2_O_3_ sheets (Fig. 2a and b) peaks compared to the bulk gallium metal (Fig. S3†). The Ga 2p_3/2_ and Ga 2p_1/2_ peaks are positioned at 1145.03 eV and 1118.13 eV respectively for bulk gallium with a shift by 0.75 eV to a higher binding energy associated with the Ga–O bonding in Ga_2_O_3_.^21^

A further understanding of the surface of the sheets was gained using surface sensitive Auger spectroscopy and corresponding depth profiling, to a depth of 40 nm (Fig. 2e). Three separate points on a single sheet were analysed with an additional control point on the silicon surface for comparison. The three separate points were chosen to represent distinct features observed on a sheet; areas 1–2 were observed to be visibly thicker as a result of wrinkling and folding of the ultrathin sheets, with an approximate thickness of ∼40 nm and area 3 as the thinnest area corresponding to a thickness of 6–7 nm and therefore there are several layers of exfoliated oxide layers in this region, as previously discussed.^47^ Depth profiling of areas 1–2 revealed gallium and oxygen on the topmost layer up to a depth of 5 nm. Interestingly, between 5–15 nm, the sheet was observed to contain predominantly gallium with no oxygen present followed by an increase in oxygen at depths between 15–35 nm. This would suggest that there is a region which contains unoxidized gallium. Depth profiling beyond this showed a decrease in both gallium and oxygen. Similarly, for area 3 as the thinnest region, gallium and oxygen are present on the uppermost layer up to a depth of ∼5 nm before a decrease in both gallium and oxygen content beyond 10 nm depth. These findings are important as they contribute towards the understanding of the mechanism of formation of these ultrathin Ga_2_O_3_ sheets, and in particular, suggests that although the thinnest layers present are completely oxidised, there also are areas of ‘sandwiched’ unoxidised gallium, specifically around regions with thickness greater than 7 nm.

**Fig. 3:**
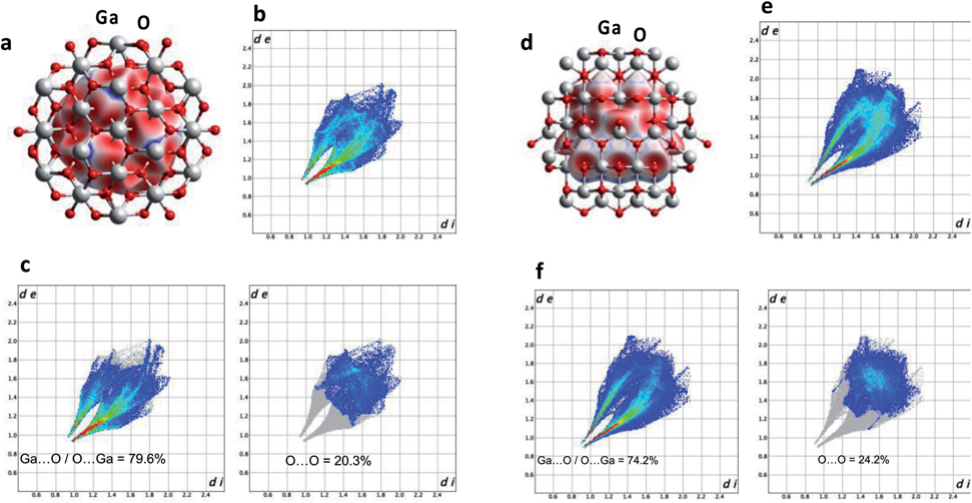
Hirshfeld surface analysis. (a and d) Hirshfeld surfaces mapped over *d*_norm_ for α- and β-Ga_2_O_3_ respectively. (b and e) Two-dimensional fingerprint plots of α- and β-Ga_2_O_3_ respectively. (c and f) Fingerprint plots delineated into Ga⋯O/O⋯Ga and O⋯O contacts for α- and β-Ga_2_O_3_ respectively.

X-ray diffraction (XRD) was used to characterize the crystal phase, crystallinity and purity of the sheets. Ga_2_O_3_ can crystallize in a number of polymorphs including α-, β-, γ-, δ- and ε phases with the α-phase typically formed as the outer oxide layer in the liquid metal.^50^ Amongst these polymorphs, the α- and β-phase are the most structurally documented with the β-phase being the most thermodynamically stable.^53^ All diffraction patterns for the Ga_2_O_3_ sheets could be indexed to α-gallium oxide (Fig. 2c) and no additional diffraction peaks of possible impurities here were detected. We attempted to relate the crystal structures of the α- and β-Ga_2_O_3_ (ICSD numbers: 27431 and 83645 respectively) to match the possible polymorph formed from the synthesized Ga_2_O_3_ sheets. α-Ga_2_O_3_ crystallizes in the high symmetry *R*3̄*c* double hexagonal close-packed system (Fig. S5†) while β-Ga_2_O_3_ crystallizes in the *C*2/*m* distorted double hexagonal close-packed system (Fig. 3b). Fingerprint plots generated from the Hirshfeld surface analysis can provide rapid visual means of discriminating the similarities and/or differences in atomic interactions between gallium and oxygen atoms, as well as summarizing the overall distribution of all close contacts quantitatively for both types of polymorph, Fig. 3b and e. This includes the variation in the percentage of Ga⋯O/O⋯Ga and O⋯O contacts in the Hirshfeld surfaces. The intermolecular contacts in both α- and β-Ga_2_O_3_ are overwhelmingly Ga⋯O/O⋯Ga (either as donor or acceptor) with 79.6% and 74.2% respectively; while the remaining contribution to the Hirshfeld surface are from O⋯O contacts at 20.3% and 24.2% respectively, Fig. 3c and f. Both polymorphs showed exceptionally small ranges of Ga⋯O/O⋯Ga contacts, evident from the two narrow bands at the lower regions of *d*_e_ and *d*_i_ on the fingerprint plot. However, the α-Ga_2_O_3_ showed more intense red and green streaks indicating that the majority of the contacts fall within the short range and can be rationalised in terms of more efficient packing amongst the atoms. O⋯O contacts are less dominant in the α-Ga_2_O_3_, as judged by the sparseness and colour of the dots in the fingerprint plot.

The thermostability of the Ga_2_O_3_ sheets was explored using thermogravimetric analysis (TGA) and differential scanning calorimetry (DSC). The TGA (Fig. 2d blue line) shows a small weight loss between 90–100 °C associated with loss of the solvent *n*-propanol physisorbed on the surface of the sheets. A slight weight increase at ∼495 °C which we predict corresponds to oxidation of gallium metal and/or subvalent gallium present sandwiched within the sheets. The DSC (Fig. 2d red line) shows a thermodynamic response associated with a phase transition at ∼875 °C which coincides with a weight increase. SEM images show that the phase transition was due to a change in morphology of the ultrathin sheets to fibres of Ga_2_O_3_ which were shown to be in the β-Ga_2_O_3_ phase (Fig. S6 and S7†).

## Applications

**Fig. 4:**
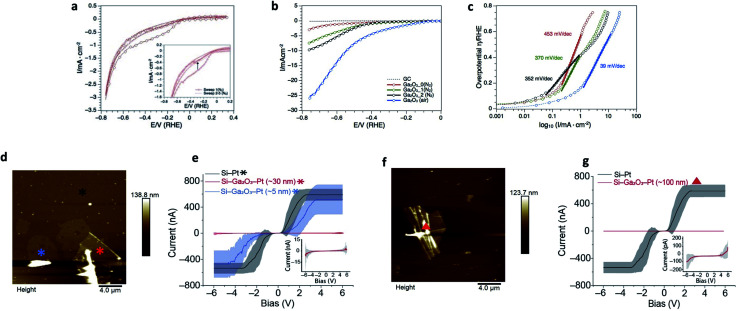
Applications of the ultrathin Ga_2_O_3_ sheets. (a–c) Electrocatalytic hydrogen evolution reaction of Ga_2_O_3_ in 0.5 M H_2_SO_4_. (a) Cyclic voltammogram traces of 70.4 μg^−1^ cm^−2^ as-prepared Ga_2_O_3_ showing stripping of surface oxygen in the first scan (inset). (b) Forward scans of 70.4 μg^−1^ cm^−2^ as-prepared Ga_2_O_3_ NS under N_2_ (red), after first (green) and second (black) conditioning cycle between −0.8 and 1.8 V *vs.* RHE. The blue CV trace represents HER of 140.8 mg^−1^ cm^−2^ Ga_2_O_3_ under atmospheric gas (air). (c) Respective Tafel slopes under different gas conditions and mass-loadings. The low Tafel slope (39 mV dec^−1^) and low overpotential (*η* (1 mA cm^−2^) = 150 mV *vs.* RHE) of Ga_2_O_3_ in this study exhibits activities toward HER which are comparable to platinum group metals on carbon support,^54^ transition metal phosphides^55^ and hybrid metal nanoparticles.^56^ (d–g) Current–potential relationship of the Ga_2_O_3_ sheets compared to silicon. (d) AFM topography image of gallium oxide sheets with thickness of 5 to 30 nm. (e) *I*–*V* measurement of Si–Ga_2_O_3_–Pt (5 nm (blue) and 30 nm (red)), and Si–Pt (black) junctions. Inset in (e) corresponds to 30 nm Ga_2_O_3_ sheet's *I*–*V* curve, in scale of nA for current. (f) AFM topography image of Ga_2_O_3_ wrinkled area with ∼100 nm thickness. (g) *I*–*V* measurement compered between Si–Ga_2_O_3_–Pt (∼100 nm (red)) and Si–Pt (black) junctions. Inset in (g) corresponds to 100 nm Ga_2_O_3_ sheet's *I*–*V* curve, in scale of pA for current. The bias is applied to the surface with bias sweep set between −6 to 6 V. *, ▲, are the location where the *I*–*V* curves were collected. Statistics were obtained by measuring the IVs at different locations around the marks in an area of 1 μm^2^. Lines are the average of 50 curves collected from each location.

Ga_2_O_3_ nanostructures have been shown to be efficient supports in photocatalytic water splitting and CO_2_ reduction.^57–61^ However, little is known about the electrocatalytic hydrogen evolution reaction (HER).^62^ Herein we report the catalytic activity of the Ga_2_O_3_ nanosheets toward electrochemical HER in 0.5 M H_2_SO_4_. The recorded cyclic voltammogram traces and calculated Tafel slopes are shown in Fig. 4a–c. The as prepared Ga_2_O_3_ nanostructures on the glassy carbon (GC) electrode revealed under protective gas (N_2_) a broad reduction event between −0.1 and −0.6 volts *vs.* RHE during the first cyclic voltammetry scan, which disappears during the subsequent numbers of sweeps (Fig. 4a, inset). The broad cathodic reduction during the first scan was reproduced when the same nanostructures were conditioned between −0.8 and 1.8 volts *vs.* RHE (Fig. S8†) and studied again under cathodic potentials. Since no dissolved oxygen was present, the first scan event is associated with stripping of the oxide layer that is regenerated under anodic potentials. The oxide layer remains stable during the subsequent conditioning cycles suggesting that the gallium nanosheets react with water under oxidative potentials (Fig. S8†). Further studies under oxygen-free and ambient conditions showed that the oxide layer plays a crucial role during the electrocatalytic activity of HER. When replacing the nitrogen by atmospheric gas (air) and increasing the mass loading, the Ga_2_O_3_ nanosheets achieved the highest activity toward HER (Fig. 4b). The calculated Tafel slope of 39 mV dec^−1^ (Fig. 4c) over a wide range of overpotential, 150–600 mV *vs.* RHE, reveals high production rates of H_2_ and suggests that Heyrovsky and Tafel determining rate steps are involved in the mechanism.^63^ This is likely due to the absorbed protons by surface oxygen since the highest hydrogen production rate appears also in the potential range where stripping of the oxygen layer occurs (Fig. 4a). The role of surface oxygen as active sites to promote the hydrogen evolution reaction has also been shown in photocatalytic water splitting on Ga_2_O_3_ structures.^61^ Overall, the obtained Tafel slope compares well with reported 2D metal oxide nanostructures where oxygen defects have been induced, such as WO_3−*x*_,^64–66^ P-MoO_3−*x*_ ^67^ and δ-MnO_2_.^68,69^ However, it is noteworthy that the required overpotentials in this study are comparably higher than that reported for 2D metal oxide structures.

Functional oxides, mainly SiO_2_, are fundamental to modern microelectronics as high-quality insulators, electroluminescent and electrochromic materials, amongst other applications. Considerable effort over several decades has resulted in high quality insulating materials now ubiquitously used in complementary metal oxide semiconductor devices. To really push these technologies into the future, the next generation of electronic components that can handle great power loads without increasing the size of the electronics systems is needed. Modern devices incorporate few-nanometer thick layers in which electrical stress can be extreme. Therefore, in recent years much effort has been devoted to developing novel gate dielectrics as alternative to silicon oxide for miniaturised electronics. Among them, Ga_2_O_3_ is one of the most promising dielectric materials due to its wide bandgap (∼4.8 eV) and excellent chemical/thermal stability. Here, we have studied the insulating electrical properties of the fabricated Ga_2_O_3_ sheets *via* conducting atomic force microscopy by wiring Ga_2_O_3_ sheets of different thicknesses. A highly doped silicon surface was used as the base electrode and a platinum electrode as the tip electrode and the fabricated thin layers of Ga_2_O_3_ in between. Results show that the current is reduced by over three orders of magnitude (Fig. 4d–g) when the thickness of the Ga_2_O_3_ layer reaches 100 nm (ESI Table S1†). Currents comparable to a bare Pt–Si junction are only observed above 6 V for a Ga_2_O_3_ layer of 30 nm thick (Fig. 4 inset in (e)). This translates to a breakdown voltage of >0.2 V nm^−1^ for the fabricated Ga_2_O_3_ layers which is comparable to highly insulating materials such as SiO*_x_* (∼0.4 V nm^−1^) that are commonly used as the insulating gate electrodes in metal–oxide–semiconductor field-effect transistor.^70,71^ This is also consistent with recent studies demonstrating Ga_2_O_3_ as an effective gate electrode in transistors.^72–74^

## Conclusions

We have established a significant advancement in the area of 2D-nanomaterials, with the development of a simple and robust method for the exfoliation of ultrathin Ga_2_O_3_ nanosheets with an average thickness of ∼5–6 nm, directly from gallium metal. The simple and benign method generates the 2D material in ∼60% yield, which is dramatically higher than has been achieved using other methods, and it avoids the use of high molecular weight solvents, surfactants and chemical stabilisers. We have also ensured scalability of the process is addressed at the inception of the science for industry uptake, with the processing under continuous flow. We envisage that this method can be extended to other low melting point metals and metalloids, for fabricating analogous layered and non-layered nanomaterials. The ability to exfoliate the oxide layer from gallium metal suggests that there must be some tangential force associated with lift mechanism in overcoming the centrifugal force in the rotating tube which would otherwise pin the high-density oxide to the surface of thin film of gallium metal. Both the tangential force and lift mechanism is consistent with the presence of micron/sub-micron spinning top topological fluid flow in the VFD. This is one of the topological fluid flows in the general model of fluid flow in the VFD which accounts for all the processing outcomes of the device, depending on the nature of the liquid and rotational speed, at a tilt angle of 45°.^49^ In addition, the ability to prepare highly insulating gallium oxide sheets has potential in device technology, and the catalytic prowess of the ultrathin sheets shows promise for application in water splitting in avoiding the use of precious metals of the transition metals and material for which its use as such is questionable on environmental grounds.

## Author contributions

K. V. and C. L. R. designed the experiments and wrote the manuscript. T. P. performed and optimised the experimental conditions, K. V. performed and analysed the SEM, AFM, Raman, XPS, XRD and TGA/DSC data, Z. G. performed the scanning Auger measurements, I. L. performed the Hirshfeld analysis, S. R. and N. D. performed the single particle electronic studies using conductive force microscopy, S. E. performed the hydrogen evolution reactions, J. R. G. performed the SEM/EDX measurements, C. G. performed and contributed to the analysis of AFM and Raman measurements, Q. S. and J. Z. performed the TEM and HRTEM measurements and M. R. A. contributed towards the development of experiments related to the hydrogen evolution reactions. All authors contributed towards editing of the manuscript.

## Conflicts of interest

The authors declare no competing interests.

## Supplementary Material

NA-003-D1NA00598G-s001

## References

[cit1] Kalantar-Zadeh K., Tang J., Daeneke T., O’Mullane A. P., Stewart L. A., Liu J., Majidi C., Ruoff R. S., Weiss P. S., Dickey M. D. (2019). ACS Nano.

[cit2] Daeneke T., Khoshmanesh K., Mahmood N., de Castro I. A., Esrafilzadeh D., Barrow S. J., Dickey M. D., Kalantar-Zadeh K. (2018). Chem. Soc. Rev..

[cit3] Messalea K. A., Carey B. J., Jannat A., Syed N., Mohiuddin M., Zhang B. Y., Zavabeti A., Ahmed T., Mahmood N., Gaspera E. D., Khoshmanesh K., Kalantar-Zadeh K. (2018). Nanoscale.

[cit4] Geim A. K., Morozov S. V., Jiang D., Zhang Y., Dubonos S. V., Grigorieva I. V., Firsov A. A. (2004). Science.

[cit5] Novoselov K. S., Jiang D., Schedin F., Booth T. J., Khotkevich V. V., Morozov S. V., Geim A. K. (2005). Proc. Natl. Acad. Sci. U. S. A..

[cit6] Coleman J. N., Lotya M., O'Neill A., Bergin S. D., King P. J., Khan U., Young K., Gaucher A., De S., Smith R. J., Shvets I. V., Arora S. K., Stanton G., Kim H.-Y., Lee K., Kim G. T., Duesberg G. S., Hallam T., Boland J. J., Wang J. J., Donegan J. F., Grunlan J. C., Moriarty G., Shmeliov A., Nicholls R. J., Perkins J. M., Grieveson E. M., Theuwissen K., McComb D. W., Nellist P. D., Nicolosi V. (2011). Science.

[cit7] Nicolosi V., Chhowalla M., Kanatzidis M. G., Strano M. S., Coleman J. N. (2013). Science.

[cit8] Hanlon D., Backes C., Doherty E., Cucinotta C. S., Berner N. C., Boland C., Lee K., Harvey A., Lynch P., Gholamvand Z., Zhang S., Wang K., Moynihan G., Pokle A., Ramasse Q. M., McEvoy N., Blau W. J., Wang J., Abellan G., Hauke F., Hirsch A., Sanvito S., O'Regan D. D., Duesberg G. S., Nicolosi V., Coleman J. N. (2015). Nat. Commun..

[cit9] Lee C., Yan H., Brus L. E., Heinz T. F., Hone J., Ryu S. (2010). ACS Nano.

[cit10] Liu K.-K., Zhang W., Lee Y.-H., Lin Y.-C., Chang M.-T., Su C.-Y., Chang C.-S., Li H., Shi Y., Zhang H., Lai C.-S., Li L.-J. (2012). Nano Lett..

[cit11] Peng J., Pan Y., Yu Z., Wu J., Zhou Y., Guo Y., Wu X., Wu C., Xie Y. (2018). Angew. Chem., Int. Ed..

[cit12] Wang Q., O'Hare D. (2012). Chem. Rev..

[cit13] Smith R. J., King P. J., Lotya M., Wirtz C., Khan U., De S., O'Neill A., Duesberg G. S., Grunlan J. C., Moriarty G., Chen J., Wang J., Minett A. I., Nicolosi V., Coleman J. N. (2011). Adv. Mater..

[cit14] Sachdev H. (2015). Science.

[cit15] Bianco E., Butler S., Jiang S., Restrepo O. D., Windi W., Goldberger J. E. (2013). ACS Nano.

[cit16] Guo X., Zhang L., Ding Y., Goodenough J. B., Yu G. (2019). Energy Environ. Sci..

[cit17] Iqbal N., Khan I., Yamani Z. H., Qurashi A. (2016). Sci. Rep..

[cit18] Alsaif M. M. Y. A., Pillai N., Kuriakose S., Walia S., Jannat A., Xu K., Alkathiri T., Mohiuddin M., Daeneke T., Kalantar-Zadeh K., Ou J. Z., Zavabeti A. (2019). ACS Appl. Nano Mater..

[cit19] Dickey M. D. (2017). Adv. Mater..

[cit20] Carey B. J., Ou J. Z., Clark R. M., Berean K. J., Zavabeti A., Chesman A. S. R., Russo S. P., Lau D. W. M., Xu Z.-Q., Bao Q., Kavehei O., Gibson B. C., Dickey M. D., Kaner R. B., Daeneke T., Kalantar-Zadeh K. (2017). Nat. Commun..

[cit21] Zhang X., Huang H., Zhang Y., Liu D., Tong N., Lin J., Chen L., Zhang Z., Wang X. (2018). ACS Omega.

[cit22] Kochat V., Samanta A., Zhang Y., Bhowmick S., Manimunda P., Asif S. A. S., Stender A. S., Vajtai R., Singh A. K., Tiwary C. S., Ajayan P. M. (2018). Sci. Adv..

[cit23] Syed N., Zavabeti A., Ou J. Z., Mohiuddin M., Pillai N., Carey B. J., Zhang B. Y., Datta R. S., Jannat A., Haque F., Messalea K. A., Xu C., Russo S. P., McConville C. F., Daeneke T., Kalantar-Zadeh K. (2018). Nat. Commun..

[cit24] Kumbhakar P., Gowda C. C., Mahapatra P. L., Mukherjee M., Malviya K. D., Chaker M., Chandra A., Lahiri B., Ajayan P. M., Jariwala D., Singh A., Tiwary C. S. (2021). Mater. Today.

[cit25] Hinterding R., Feldhoff A. (2019). Z. Phys. Chem..

[cit26] Mei J., Liao T., Kou L., Sun Z. (2017). Adv. Mater..

[cit27] Jones D. B., Chen X., Sibley A., Quinton J. S., Shearer C. J., Gibson C. T., Raston C. L. (2016). Chem. Commun..

[cit28] Al-Antaki A. H. M., Luo X., Alharbi T. M. D., Harvey D. P., Pye S., Zou J., Lawrance W., Raston C. L. (2019). RSC Adv..

[cit29] Batmunkh M., Vimalanathan K., Wu C., Bati A. S. R., Yu L., Tawfik S. A., Ford M. J., Macdonald T. J., Raston C. L., Priya S., Gibson C. T., Shapter J. G. (2019). Small Methods.

[cit30] Vimalanathan K., Gascooke J., Suarez-Martinez I., Marks N., Kumari H., Garvey C., Atwood J., Lawrance W., Raston C. L. (2016). Sci. Rep..

[cit31] Alharbi T. M. D., Vimalanathan K., Lawrance W. D., Raston C. L. (2018). Carbon.

[cit32] Britton J., Stubbs K. A., Weiss G. A., Raston C. L. (2017). Chem.–Eur. J..

[cit33] Totoiu C. A., Phillips J. M., Reese A. T., Majumdar S., Girguis P. R., Raston C. L., Weiss G. A. (2020). PLoS One.

[cit34] Phillips J. M., Ahamed M., Duan X., Lamb R. N., Qu X., Zheng K., Zou J., Chalker J. M., Raston C. L. (2019). ACS Appl. Bio Mater..

[cit35] Luo X., Al-Antaki A. H. M., Vimalanathan K., Moffatt J., Zheng K., Zou Y., Zou J., Duan X., Lamb R. N., Wang S., Li Q., Zhang W., Raston C. L. (2018). React. Chem. Eng..

[cit36] Pye S. J., Dalgarno S. J., Chalker J. M., Raston C. L. (2018). Green Chem..

[cit37] Heine V. (1968). J. Phys. C: Solid State Phys..

[cit38] Robinson G. H. (1999). Acc. Chem. Res..

[cit39] Ghigna P., Spinolo G., Parravicini G. B., Stella A., Migliori A., Kofman R. (2007). J. Am. Chem. Soc..

[cit40] Gao Y., Bando Y. (2002). Nature.

[cit41] Qin B., Schneide U. (2016). J. Am. Chem. Soc..

[cit42] Spells K. E. (1936). Proc. Phys. Soc., London.

[cit43] Zavabeti A., Ou J. Z., Carey B. J., Syed N., Orrell-Trigg R., Mayes E. L. H., Xu C., Kavehei O., O'Mullane A. P., Kaner R. B., Kalantar-Zadeh K., Daeneke T. (2017). Science.

[cit44] Syed N., Zavabeti A., Mohiuddin M., Zhang B., Wang Y., Datta R. S., Atkin P., Carey B. J., Tan C., van Embden J., Chesman A. S. R., Ou J. Z., Daeneke T., Kalantar-Zadeh K. (2017). Adv. Funct. Mater..

[cit45] Tang S.-Y., Qiao R., Lin Y., Li Y., Zhao Q., Yuan D., Yun G., Guo J., Dickey M. D., Huang T. J., Davis T. P., Kalantar-Zadeh K., Li W. (2019). Adv. Mater. Technol..

[cit46] Vimalanathan K., Chen X., Raston C. L. (2014). Chem. Commun..

[cit47] Vimalanathan K., Shrestha R. G., Zhang Z., Zou J., Nakayama T., Raston C. L. (2017). Angew. Chem., Int. Ed..

[cit48] Luo X., Smith P., Raston C. L., Zhang W. (2016). ACS Sustainable Chem. Eng..

[cit49] Alharbi T. M. D., Jellicoe M., Luo X., Vimalanathan K., Alsulami I. K., Al Harbi B. S., Igder A., Alrashaidi F. A. J., Chen X., Stubbs K. A., Chalker J. M., Zhang W., Boulos R. A., Jones D. B., Quinton J. S., Raston C. L. (2021). Nanoscale Adv..

[cit50] Regan M. J., Tostmann H., Pershan P. S., Magnussen O. M., DiMasi E., Ocko B. M., Deutsch M. (1997). Phys. Rev. B: Condens. Matter Mater. Phys..

[cit51] Creighton J. A., Withnall R. (2000). Chem. Phys. Lett..

[cit52] Wundrack S. , Pakdehi D. M., Dempwolf W., Schmidt N., Pierz K., Michaliszyn L., Spende H., Schmidt A., Schumacher H. W., Stosch R. and Bakin A., 2019, arXiv:Materials Science

[cit53] Bosi M., Mazzolini P., Seravalli L., Fornari R. (2020). J. Mater. Chem. C.

[cit54] Yang J., Chen B., Liu X., Liu W., Li W. Z., Dong J., Chen W., Yan W., Yao T., Duan X., Wu Y., Li Y. (2018). Angew. Chem., Int. Ed..

[cit55] Wang D., Zhang X., Zhang D., Shen Y., Wu Z. (2016). Appl. Catal., A.

[cit56] Amin M. A., Ahmed E. M., Mostafa N. Y., Alotibi M. M., Darabdhara G., Das M. R., Wysocka J., Ryl J., El-Rehim S. S. A. (2016). ACS Appl. Mater. Interfaces.

[cit57] Busser G. W., Mei B., Weide P., Vesborg P. C. K., Stuhrenberg K., Bauer M., Huang X., Wilinger M.-G., Chorkendorff I., Schlogl R., Muhler M. (2015). ACS Catal..

[cit58] Yanagida T., Sakata Y., Imamura H. (2004). Chem. Lett..

[cit59] Wang X., Xu Q., Li M., Shen S., Wang X., Wang Y., Feng Z., Shi J., Han H., Li C. (2012). Angew. Chem., Int. Ed..

[cit60] Yamamoto M., Yoshida T., Yamamoto N., Nomoto T., Yamamoto Y., Yagi S., Yoshida H. (2015). J. Mater. Chem. A.

[cit61] Wang Y., Zhuang P., Yue H., Dong H., Zhou X. (2019). J. Phys. Chem. C.

[cit62] Kakoria A., Devi B., Anand A., Halder A., Koner R. R., Sinha-Ray S. (2019). ACS Appl. Nano Mater..

[cit63] Shinagawa T., Garcia-Esparza A. T., Takanabe K. (2015). Sci. Rep..

[cit64] Li Y. H., Liu P. F., Pan L. F., Wang H. F., Yang Z. Z., Zheng L. R., Hu P., Zhao H. J., Gu L., Yang H. G. (2015). Nat. Commun..

[cit65] Sharma L., Kumar P., Halder A. (2019). ChemElectroChem.

[cit66] Zheng T., Sang W., He Z., Wei Q., Chen B., Li H., Cao C., Huang R., Yan X., Pan B., Zhou S., Zeng J. (2017). Nano Lett..

[cit67] Li L., Zhang T., Yan J., Cai X., Liu S. (2017). Small.

[cit68] Zhao Y., Chang C., Teng F., Zhao Y., Chen G., Shi R., Waterhouse G. I. N., Huang W., Zhang T. (2017). Adv. Energy Mater..

[cit69] Zhu Y., Lin Q., Zhong Y., Tahini H. A., Shao Z., Wang H. (2020). Energy Environ. Sci..

[cit70] Ponomarenko L. A., Gorbachev R. V., Yu G. L., Elias D. C., Jalil R., Patel A. A., Mishchenko A., Mayorov A. S., Woods C. R., Wallbank J. R., Mucha-Kruczynski M., Piot B. A., Potemski M., Grigorieva I. V., Novoselov K. S., Guinea F., Fal'ko V. I., Geim A. K. (2013). Nature.

[cit71] Yang H., Withers F., Gebremedhn E., Lewis E., Britnell L., Felten A., Palermo V., Haigh S., Beljonne D., Casiraghi C. (2014). 2D Mater..

[cit72] Joshi C., Zhang Y., Xia Z., Sun W., Arehart A. R., Ringel S., Lodha S., Rajan S. (2019). IEEE Electron Device Lett..

[cit73] Kamimura T., Nakata Y., Higashiwaki M. (2021). Jpn. J. Appl. Phys..

[cit74] Wong M. H. (2019). IEEE Electron Device Lett..

