# Supplementary material for: High shear in situ exfoliation of 2D gallium oxide sheets from centrifugally derived thin films of liquid gallium
Source: Nanoscale Adv. 2021 Sep 1;3(20):5785–92. doi: 10.1039/d1na00598g (PMC9419649; doi:10.1039/d1na00598g)
Supplement: NA-003-D1NA00598G-s001 [file NA-003-D1NA00598G-s001.pdf]

Electronic Supplementary Material (ESI) for Nanoscale Advances.
This journal is © The Royal Society of Chemistry 2021

Supplementary information

High shear *in situ* exfoliation of 2D gallium oxide sheets from centrifugally derived thin films of liquid gallium

Kasturi Vimalanathan[1]*, Timotheos Palmer[1], Zoe Gardner[1], Irene Ling[2], Soraya Rahpeima[1,3], Sait Elmas[1], Jason R. Gascooke[1], Christopher T. Gibson[1,4], Qiang Sun[5,6], Jin Zou[5,6], Mats R. Andersson[1], Nadim Darwish[3] and Colin L. Raston[1]*

[1] Flinders Institute for Nanoscale Science and Technology, College of Science and Engineering, Flinders University, Adelaide SA 5001, Australia

[2] School of Science, Monash University Malaysia, Jalan Lagoon Selatan, Bandar Sunway, 47500 Selangor, Malaysia

[3] School of Molecular and Life Sciences, Curtin Institute for Functional Molecule and Interfaces, Curtin University, Bentley, Western Australia 6102, Australia

[4] Flinders Microscopy and Microanalysis, College of Science and Engineering, Flinders University, Bedford Park, SA 5042, Australia

[5] Centre for Microscopy and Microanalysis, The University of Queensland, Brisbane, QLD 4072, Australia

[6] Materials Engineering, The University of Queensland, Brisbane, QLD 4072, Australia.

*Corresponding authors email: kasturi.vimalanathan@flinders.edu.au and colin.raston@flinders.edu.au

Experimental method

1. **Experimental Section**

Synthesis of the Ga_2O_3 sheets Gallium solid was crushed in a mortar and pestle to finer powder particles prior to immersing in 1 mL of *n*-propanol (10 mg/mL). The mixture was then heated on a hot plate to *ca* 30 °C for ~ 60 seconds to achieve a molten liquid which was then subsequently transferred to a VFD quartz tube (20 mm OD, 17.5 mm ID, 18.5 cm long). The VFD process was optimised by systematically varying the rotational speed (4000 rpm to 9000 rpm) and the inclination angle (0° to 90°), with the optimised conditions 7000 rpm and θ 45^0 respectively. The molten gallium was then processed for a residence time of ~30 minutes, following centrifugation (g=3.22) of the post processed sample for 30 minutes to remove any residual bulk gallium and contamination present. The method was then translated into a continuous flow processing, with a jet feed set (flow rate 1.0 mL/min) to deliver *n*-propanol into the rapidly rotating tube containing the molten gallium. The ultrathin sheets of Ga_2O_3 were then collected and characterized.

Material characterizations Morphology, structure and surface analysis of the samples were characterized by scanning electron microscopy (SEM, FEI F50) equipped with energy dispersive X-ray spectroscopy (EDX), XRD patterns were obtained using an X-ray diffractometer (Bruker, Germany) using Co Kα radiation (λ = 1.78892 Å). AFM height images were acquired using a Bruker Multimode 8 AFM with a Nanoscope V controller using tapping mode in air, with all parameters including set-point, scan rate, and feedback gains adjusted to optimize image quality. Raman spectra were acquired using a Witec alpha300R Raman microscope at an excitation laser wavelength of 532 nm with a 100X objective (numerical aperture 0.90). The scanning Auger analysis was conducted with a PHI 710 Scanning Auger Nanoprobe. Ar^+ ions were used in the depth profiling for etching the sheets. PerkinElmer STA 8000 was used to evaluate the thermal stability of the samples under nitrogen atmosphere. The heating process was performed at 10 ºC/min increase rate, under nitrogen atmosphere. TEM and HRTEM (JEOL JEM-2100F, operated at 200 kV and equipped with an EDS detector) samples were prepared by drop-casting the dispersion onto standard holey carbon grids. The chemical composition of the prepared samples characterized using XPS with an X-ray source producing 1253.6 eV (Mg Kα radiation) and 200 W power using a SPECS PHOIBOS-HSA300 hemispherical analyser (Berlin, Germany). All XPS spectra were analysed with Casa XPS using a Shirley background.

2. **<u>Additional Characterization/Equipment Details</u>**

<u>Raman Spectroscopy and Microscopy</u>

Raman spectra were acquired using a Witec alpha300R Raman microscope at an excitation laser wavelength of 532 nm with a 100X objective (numerical aperture 0.90). Typical integration times for single Raman spectra were typically 30 s and averaged from 2 to 3 repetitions. Confocal Raman images were acquired with integrations typically 3 to 5 seconds per pixel. Each pixel in the Raman images represents a Raman spectrum with the number of pixels in a typical Raman image representing hundreds to thousands of spectra. Confocal Raman images are generated by plotting the intensity of a specified region of each Raman spectrum that corresponds to a material, versus the X-Y position of the excitation laser as it scans the sample surface.

<u>Atomic Force Microscopy</u>

AFM images were acquired using a Bruker Multimode 8 AFM with a Nanoscope V controller using tapping mode in air, with all parameters including set-point, scan rate, and feedback gains adjusted to optimize image quality. The AFM probes used were Mikromasch HQ:NSC15 Si probes with a nominal spring constant of 40 N/m and a nominal tip diameter of 16 nm. The scanner was calibrated in the x, y, and z directions using silicon calibration grids (Bruker model numbers VGRP: 10 µm pitch, 180 nm depth, PG: 1 µm pitch, 110 nm depth). Analysis of AFM images was performed using Nanoscope analysis software version 1.4. Presented AFM topography images were flattened, and the cross sections acquired using the section tool in the Nanoscope analysis software.

<u>Scanning Auger Spectroscopy</u>

The AES analysis was conducted with a PHI 710 Scanning Auger Nanoprobe. The Ga_2O_3 sheets were drop-casted onto silicon wafers which were used for the characterisation. Ar^+ ions were used in the depth profiling for etching the sheets. The Scanning Auger Nanoprobe parameters were as follows: etching speed, 0.24 nm s^{-1}; ion voltage, 2kV; interval; 6 s; depth; 40 nm; and scanning area of 0.19µm. Silicon dioxide was as a standard for the etching speed determination. The Scanning Electron Microscopy on the Scanning Auger Nanoprobe used a current intensity of 10 nA and an acceleration voltage of 10kV. Ga_{LMM}, O_{KLL} and Si_{LVV} Auger lines were used for plotting the depth profiles of each sample.

3. **Applications Details**

<u>Conductive atomic force microscopy measurements</u>

Conductive- atomic force microscopy (C-AFM) was used for imaging and dc conductivities. Current-voltage (I−V) data were collected by a Bruker ICON head with peak force Tuna module at room temperature using conductive platinum tip (RMN-25PT300B) having spring constant of 18 N.m-1. The image resolution, scan rate and peak force set point were set to 256 points, 1 Hz and 50 nN, respectively. Here, n-type (phosphorus doped) single-side polished Si wafer with the thickness of 500-550 µm and (111) ± 0.05° (with resistivity of <0.002 Ω.cm) was used.

Electrocatalytic hydrogen evolution reaction (HER) measurements

Electrocatalytic hydrogen evolution reactions (HER) were performed on an AUTOLAB potentiostat (Metrohm AG, Switzerland) using a glassy carbon (GC) electrode (0.071 cm^2) as working (WE), a platinum wire as counter (CE) and Ag|AgCl (1 M KCl) as reference electrode (RE) in 0.5M H_2SO_4 as electrolyte solution. The glassy carbon electrode was polished with 0.3 µm Alumina (Micro Polish, IONODE) prior to changing the mass loading. Here, 5 or 10 µL of 1 mg/mL Ga_2O_3 nanostructures in n-propanol were drop-casted onto the WE, dried under ambient temperature for 3 h and tested under oxygen-free and atmospheric gas conditions using N_2 as protective gas. This equals to 70.4 and 140.8 $\mu g/cm^{-2}$ mass loading of Ga_2O_3 electrocatalyst. Higher mass-loadings showed leaching of the catalysts from the glassy carbon disc once dispersed in 0.5 M H_2SO_4 electrolyte solution. Hence, for the sake of reproducibility and to avoid manipulation of the geometric surface of the GC working electrode, only 1 or 2 droplets of electrocatalyst (5 and 10 µL) were drop-casted onto the GC surface. All recorded working potentials vs. Ag|AgCl (1M KCl) were converted into the reversible hydrogen electrode (RHE) according to the equation $E_{(RHE)} = E_{(Ag|AgCl)} + 0.059 \bullet pH + E^0_{(Ag|AgCl)}$, where $E^0_{(Ag|AgCl,\ 1M\ KCl)} = 0.235$ V

Supplementary Figure 1

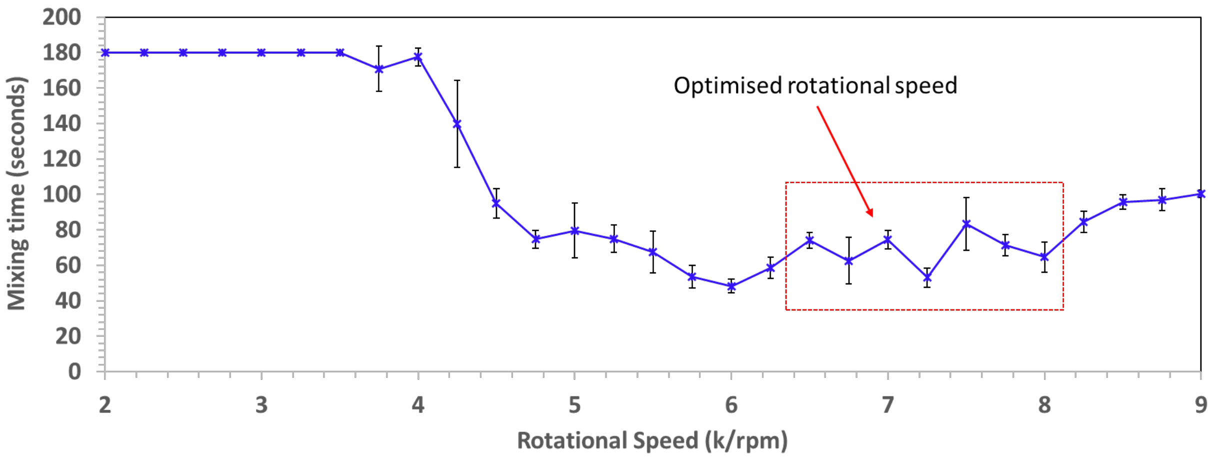

Fig. S1 Mixing times of the solvent in the VFD at the different rotational speeds were measured to identify the optimised speed for the processing of liquid gallium. 1mL of *n*-propanol was placed in the VFD operating at an inclination angle of 45° and varying the rotational speed (2000–9000 rpm) and the time taken for a drop of dye to uniformly mix with the bulk liquid was measured in seconds (all measurements were carried out in triplicates).

Supplementary Figure 2

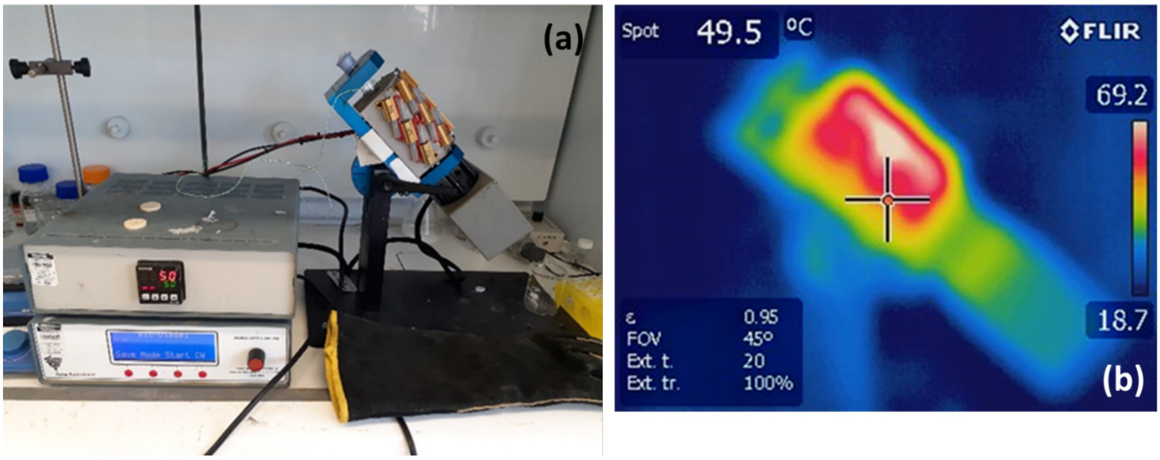

Fig. S2 (a) Control experiment using a 'plug and play' heating jacket as an alternative to heating on a hotplate and (b) Thermal imaging of the VFD with the heating jacked showing a temperature of 50 ^{0}C.

Supplementary Figure 3

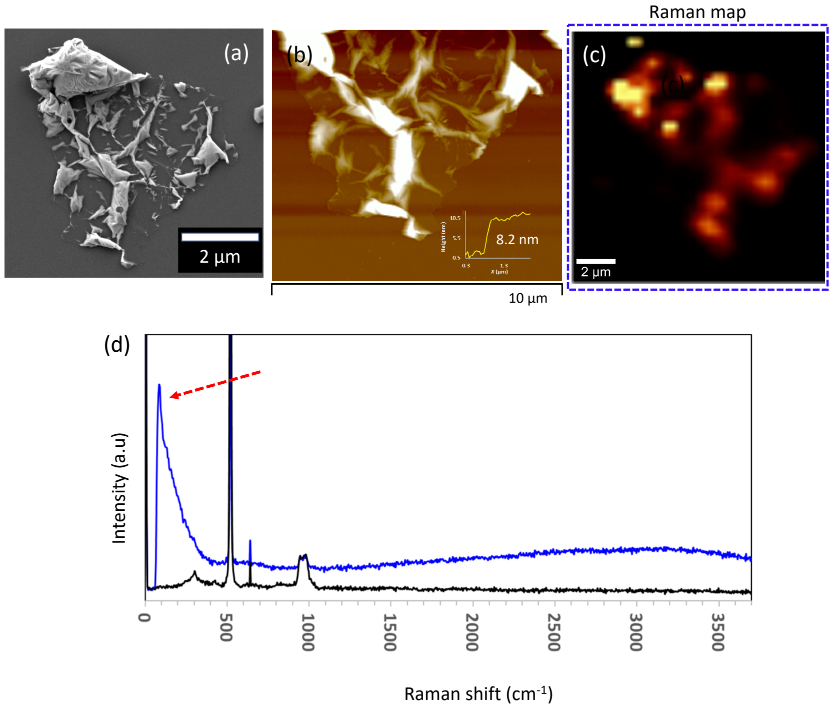

Fig. S3 Raman analysis of a Ga_2O_3 sheet; **a-b** SEM and AFM height image of a Ga_2O_3 sheet; **c** Raman map of the sheet imaged in (a-b) and **d** The Raman spectra that corresponds to the map in (c) mapping the peak labelled with the red arrow, ~50-100 cm^{-1}

Supplementary Figure 4

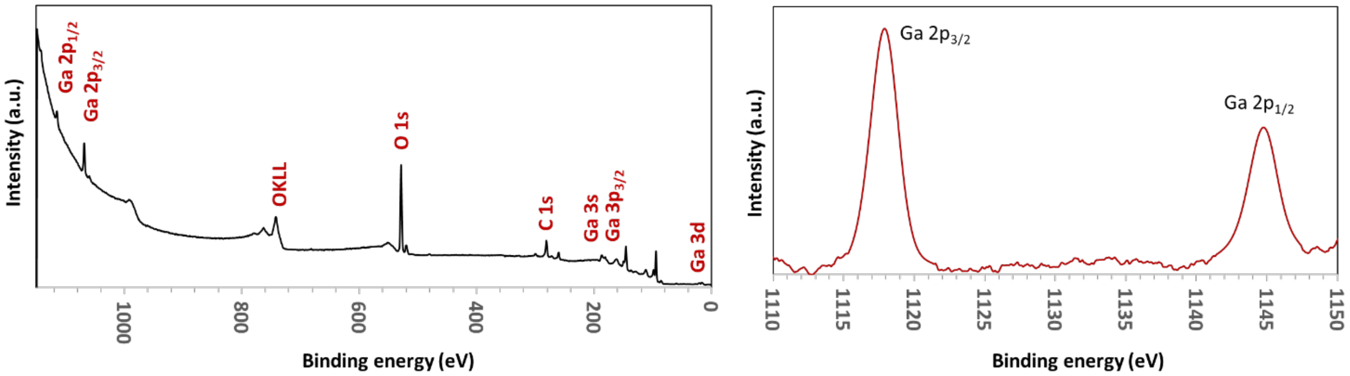

Fig. S4: XPS survey spectra of bulk gallium metal, and (b) High resolution spectra of the doublet 2p region

Supplementary Figure 5

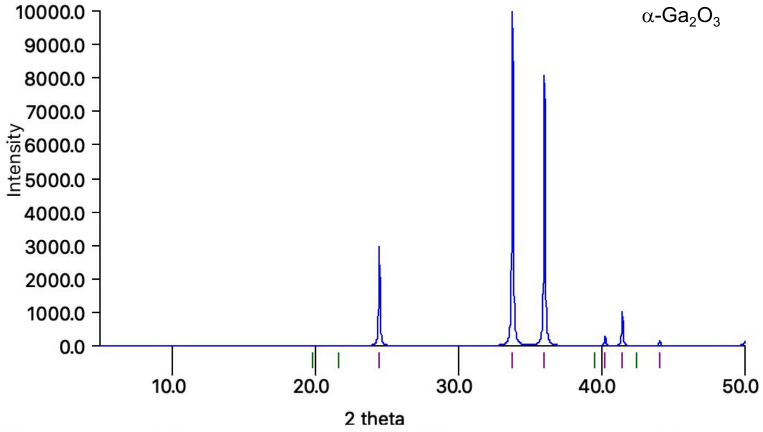

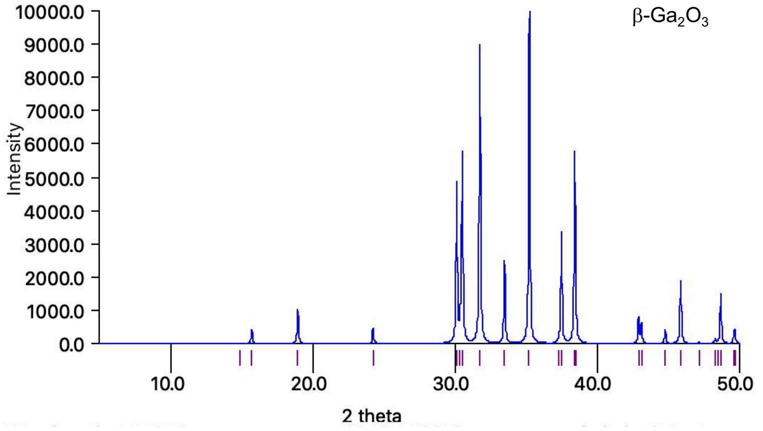

Fig. S5: XRD spectra for α- and β-Ga_2O_3. Spectra were computed using Mercury software using the crystal structures of the α- and β-Ga_2O_3 (ICSD numbers: 27431 and 83645 respectively).

Supplementary Figure 6

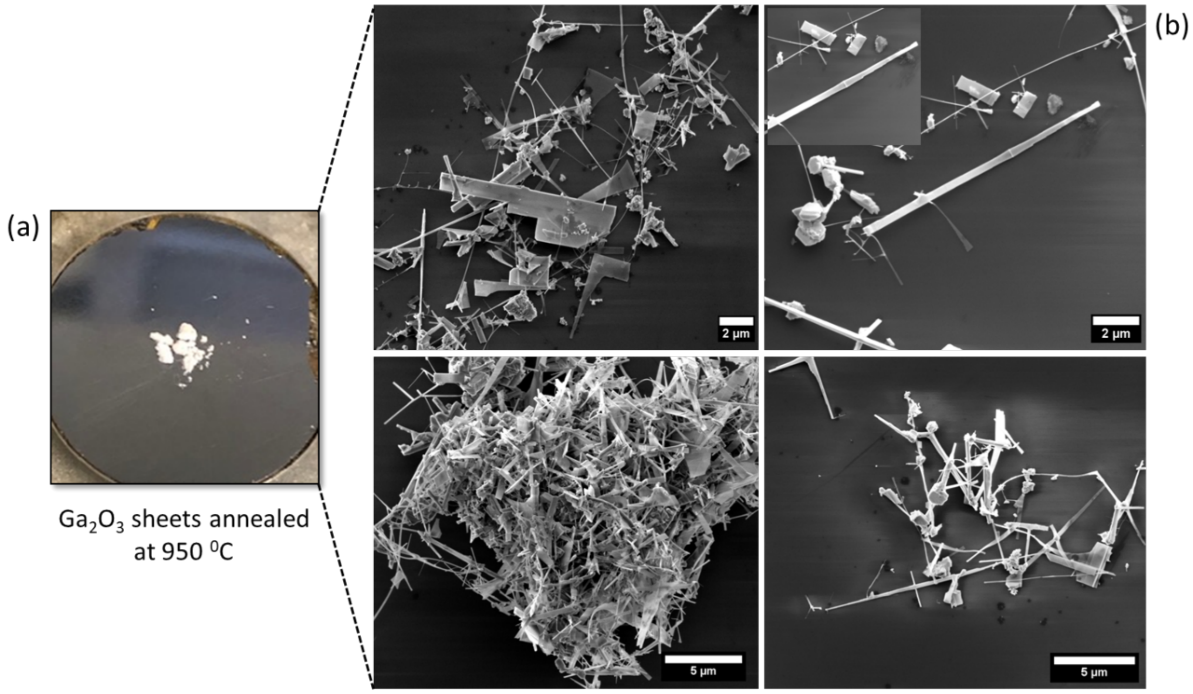

Fig. S6: Characterization of material after thermal analysis. TGA/DSC was carried out at 10 ^{0}C min^{-1} of temperature increase under N_2. (a) A white powder was collected after heating at 950 ^{0}C and (b) SEM images of gallium oxide fibres/scrolls formed after heating at 950 ^{0}C.

Supplementary Figure 7

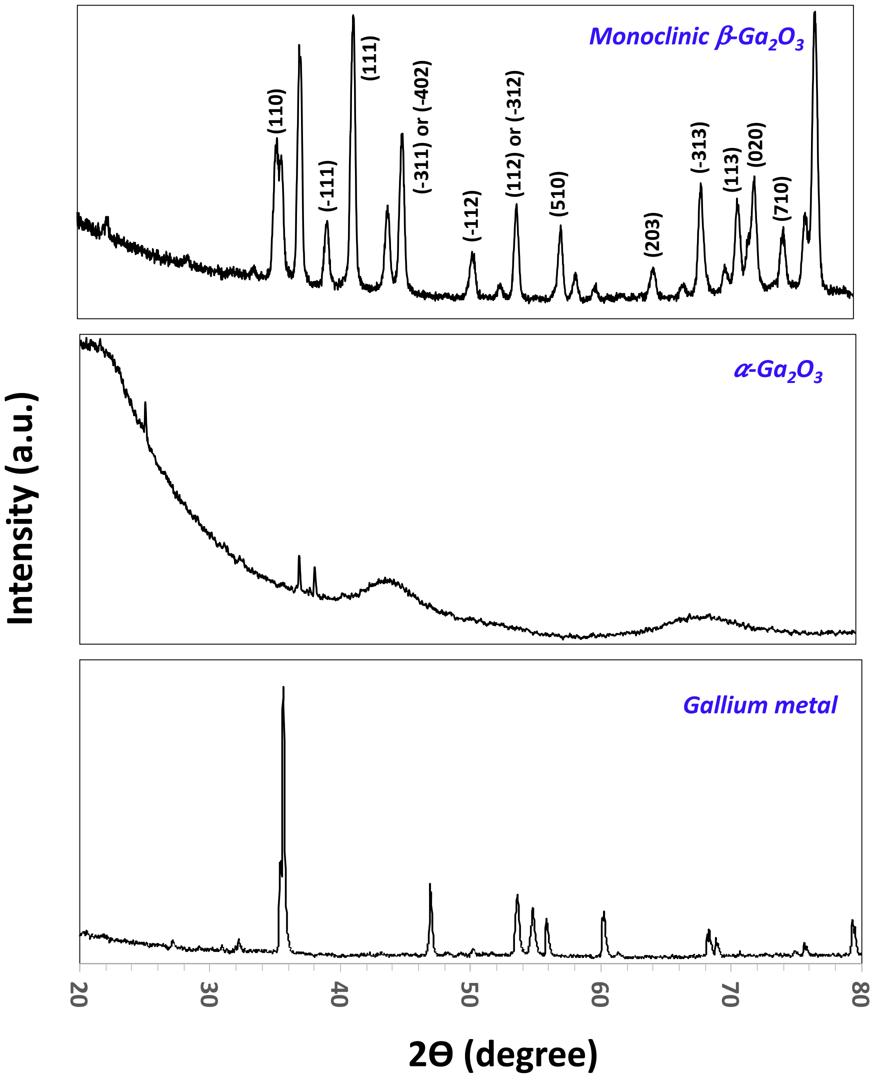

Fig. S7: XRD analysis of the gallium oxide fibres/scrolls formed after heating at 950 ^{0}C.

Supplementary Figure 8

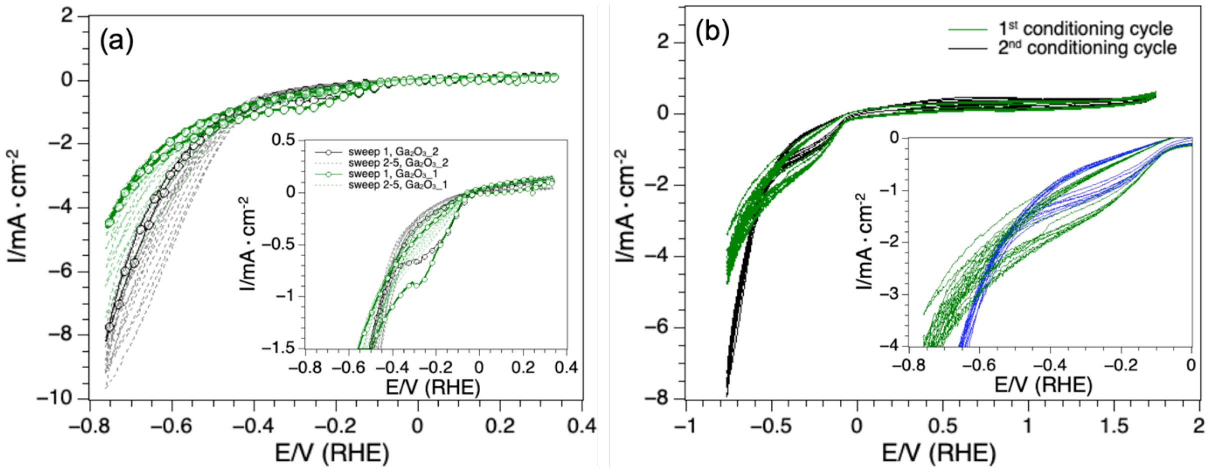

Fig. S8: Recorded cyclic voltammogram (CV) traces of HER on Ga_2O_3 nanosheets after 1st and 2nd conditioning cycles; (b) Recorded CV traces of the conditioning cycles in the potential range -0.8 to 1.8 V vs. RHE. All CV traces were recorded under protective gas condition using nitrogen.

Supplementary Figure 9

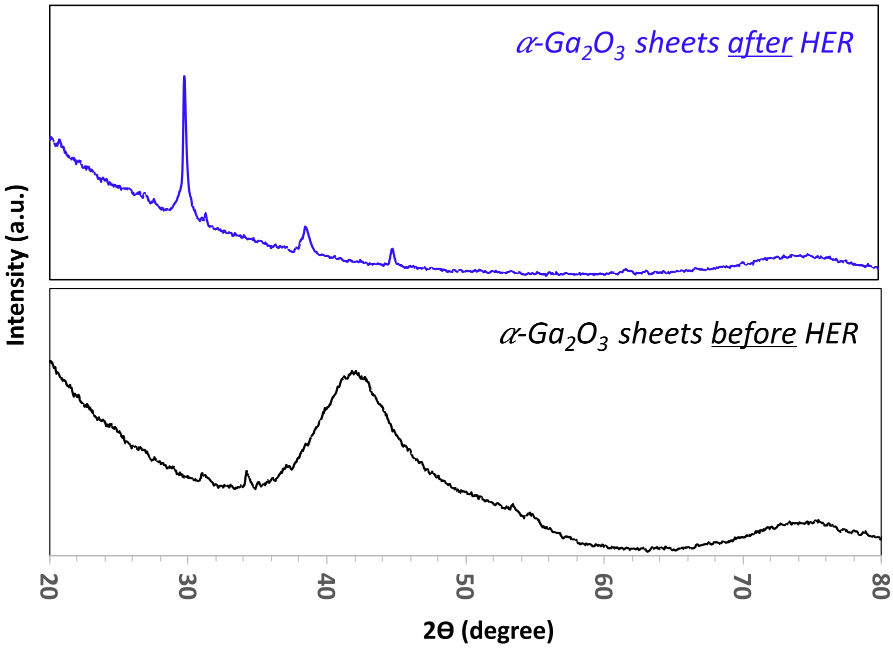

Fig. S9: XRD analysis before and after the electrocatalytic hydrogen evolution reaction (HER). The amorphous α-Ga_2O_3 sheets synthesized in the VFD were observed to exhibit crystalline characteristics after electrocatalytic treatment.

Supplementary Table 1

Thickness (nm)	Average current at 6 V (nA)
5	> 500
30	3
100	0.05-0.1

Table S1: Average current at 6V at different thicknesses of the Ga_2O_3 sheets
